# Overcoming EZH2 Inhibitor Resistance by Taxane in PTEN-Mutated Cancer

**DOI:** 10.7150/thno.34700

**Published:** 2019-07-09

**Authors:** Linlin Ma, Yuqian Yan, Yang Bai, Yinhui Yang, Yunqian Pan, Xiaokun Gang, R. Jeffrey Karnes, Jun Zhang, Qiubo Lv, Qiang Wu, Haojie Huang

**Affiliations:** 1 Department of Obstetrics and Gynecology, Beijing Hospital, National Center of Gerontology, Beijing 100730, China;; 2 Department of Biochemistry and Molecular Biology, Mayo Clinic College of Medicine and Science, Rochester, MN 55905, USA;; 3 Department of Urology, Mayo Clinic College of Medicine and Science, Rochester, MN 55905, USA;; 4 Department of Laboratory Medicine and Pathology, Mayo Clinic College of Medicine and Science, Rochester, MN 55905, USA;; 5 Department of Urology, Tongji Hospital, Tongji University School of Medicine, Shanghai 200065, China;; 6 Mayo Clinic Cancer Center, Mayo Clinic College of Medicine and Science, Rochester, MN 55905, USA.

**Keywords:** EZH2, FOXO1, PTEN, cell death, taxane, cancer

## Abstract

**Rationale**: The Polycomb group (PcG) protein EZH2 is implicated in cancer progression due to its frequent overexpression in many cancer types and therefore is a promising therapeutic target. Forkhead box transcription factor-1 (FOXO1) is a tumor suppressor that is often transcriptionally downregulated in human cancers such as prostate cancer although the underlying regulatory mechanisms remain elusive.

**Methods**: Analysis of EZH2 ChIP-seq and ChIP-on-chip data in various cell types was performed. ChIP-qPCR, RT-qPCR, and western blot analyses were conducted to determine the mechanism by which EZH2 represses FOXO1 expression. Immunohistochemistry was employed to assess the correlation between EZH2 and FOXO1 protein expression in prostate cancer patient specimens. *In vitro* MTS (3-(4,5-dimethylthiazol-2-yl)-5-(3-carboxymethoxyphenyl)-2-(4-sulfophenyl)-2H-tetrazolium) and animal experiments were performed to determine the anti-cancer efficacy of EZH2 inhibitor alone or in combination of docetaxel, a chemotherapy agent of the taxane family, and dependency of the efficacy on FOXO1 expression.

**Results**: We demonstrated that EZH2 binds to the *FOXO1* gene promoter. EZH2 represses *FOXO1* gene expression at the transcriptional level. EZH2 protein level inversely correlated with FOXO1 protein expression in prostate cancer patient specimens. This repression requires the methyltransferase activity and the functional PRC2 complex. While effectively inducing loss of viability of PTEN-positive 22Rv1 prostate cancer cells, EZH2 inhibitor failed to inhibit growth of PTEN-negative C4-2 prostate cancer cells. Co-treatment with docetaxel overcame EZH2 inhibitor resistance in PTEN-negative cancer cells *in vitro* and in mice. This effect was largely mediated by docetaxel-induced nuclear localization and activation of FOXO1.

**Conclusions**: This study identifies FOXO1 as a *bona fide* repression target of EZH2 and an essential mediator of EZH2 inhibition-induced cell death. Our findings suggest that EZH2 repression of FOXO1 can be targeted by EZH2 inhibitor as a monotherapy for PTEN-proficient cancers or in combination with taxane for treatment of cancers with PTEN mutation or deletion.

## Introduction

The Polycomb group (PcG) protein EZH2 interacts with other PcG proteins to form the Polycomb repressive complex 2 (PRC2) that plays important roles in gene silencing via catalyzing histone H3 lysine 27 trimethylation (H3K27me3) 1-4. This Polycomb-dependent (PcD) function of EZH2 is implicated in cancer due to its frequent overexpression in many aggressive, metastatic tumors such as prostate and breast cancer 5-9. The PcD activity is important for EZH2 to repress expression of tumor suppressor genes such as *p16^INK4A^*, *ADRB2*, and *DAB2IP* 10-12. A Polycomb-independent (PcI), but still methyltransferase-dependent function of EZH2 is also implicated in prostate cancer progression 13, suggesting that EZH2 is a viable therapeutic target. Indeed, a few small molecule inhibitors of EZH2, such as GSK126, have been developed to target the methyltransferase activity of EZH2 for cancer treatment 14-16. However, resistance to EZH2 enzymatic inhibitors often develops and the underlying mechanism remains poorly understood.

FOXO family proteins including FOXO1, FOXO3, FOXO4, and FOXO6 (the human orthologs of *Caenorhabditis elegans* DAF-16 and *Drosophila melanogaster* dFOXO) are often recognized as tumor suppressors 17. Activation of these factors results in transcriptional upregulation of genes involved in apoptosis (e.g., *Bim* and *FasL*), cell cycle arrest (e.g., *p27^KIP1^* and *p21^CIP1^*), and oxidative stress detoxification (e.g., *MnSOD* and *CAT*) 18-21. Notably, the *FOXO1* gene is frequently lost due to genomic deletion or transcriptional downregulation in human cancers such as prostate cancer 22, implying that it may function as a tumor suppressor during prostate tumorigenesis. In support of this notion, mouse genetic studies show that while deletion of *FOXO1* alone is insufficient to induce tumorigenesis in the mouse prostate, FOXO1 loss cooperates with TMPRSS2-ERG fusion, the most frequent genetic alteration in human prostate cancers to promote tumorigenesis 23.

Phosphatase and tension homolog (PTEN) is a well-established tumor suppressor. FOXO1 is a key downstream effector of PTEN in constraining cell growth and survival 24. PTEN loss results in activation of AKT, CDK1, and CDK2 kinases, which in turn leads to phosphorylation of FOXO1, exclusion of FOXO1 from the nucleus, and loss of the tumor suppressor functions in the nucleus 18, 25-27. The expression and activity of FOXO factors are strongly controlled by post-translational modifications such as phosphorylation, acetylation 28, methylation 29 and ubiquitination 30. Several microRNAs (miRs), including miR-96, miR-182 and miR-370, have been identified as regulators of FOXO expression in different cancer types 31-33. There are not many reports regarding the transcriptional regulation of *FOXO* genes 34, 35. While a number of mechanisms have been suggested for the repression of FOXO1 function during cancer progression 23, the mechanism underlying the transcriptional regulation of FOXO1 in cancer remains poorly understood.

In this study, we identify *FOXO1* as a novel downstream repression target gene of EZH2 and demonstrate that EZH2 repression of FOXO1 requires its methyltransferase activity and the intact PRC2 complex. We further show that PTEN-mutated cancer cells are resistant to EZH2 inhibitor-mediated killing, but the resistance can be overcome by docetaxel-induced nuclear localization of FOXO1.

## Methods

### Cell lines, cell culture and transfection

22Rv1 and LNCaP cell lines were purchased from American Type Culture Collection (ATCC) (Manassas, VA). C4-2 cells were purchased from Uro Corporation (Oklahoma City, OK). BPH-1 cells were kindly provided by Dr. Simon Hayward 36. Cells were cultured in RPMI 1640 supplemented with 10% fetal bovine serum and 100 units/ml penicillin and 100 μg/ml streptomycin. Cells were incubated at 37°C with 5% CO2. These cell lines have been tested and authenticated (karyotyping, AR expression, and PTEN mutation status) for fewer than 6 months prior to this submission. The transient transfection was performed by electroporation using an Electro Square Porator ECM 830 (BTX) as described previously 37 or by Lipofectamine™ 2000 transfection reagent (Thermo Fisher Scientific). The stable cell lines were established with lentivirus.

### Antibodies and reagents

The antibodies used are as follows: EZH2 (5246S), FOXO1 (2880S), Myc tag (2278S), cleaved caspase-3 (Asp175) (D3E9) (9579S) and cleaved PARP (ASP214) (D64E10) (5625S) were purchased from Cell Signaling Technology; Ki67 (ab15580), H3K27me3 (ab6002) and H3K27ac (ab177178) from Abcam; EZH1 (sc-515817), ERK 2 (D-2) (sc-1647) and total H3 (sc-10809) from Santa Cruz; EED (05-1327) and SUZ12 (3C1.2) (05-1320) from Millipore; β-Tubulin (DHSB E7, AB-528499) from Developmental Studies Hybridoma Bank (DSHB); E-cadherin (BD 610181) from BD Biosciences; BIM (AB17003) from Chemicon International. Plasmids for Myc-tagged wild-type EZH2 and SET domain truncated (ΔSET) EZH2 were described previously 38. The siRNA pool against human *EZH2* gene and nonspecific control siRNAs were purchased from Thermo Fisher Scientific. Lenti CRISPR v2 (sg empty vector) was purchased from Addgene. FOXO1-specific shRNA construct and negative control shRNA were purchased from Sigma-Aldrich and transfected with lentivirus. The sequences of siRNA and shRNA are listed in Table S1. The EZH2 small molecule inhibitor GSK126 was kindly provided by GlaxoSmithKline (GSK). Another EZH2 inhibitor GSK343 (HY-13500) was purchased from MedChemExpress (Monmouth Junction, NJ, USA). HDAC inhibitor suberoylanilide hydroxamic acid (SAHA) was purchased from Cayman Chemical (San Diego, CA, USA). Gene- specific generation-2.5 antisense oligonucleotides (ASO) for human EZH2 (633365), for mouse Ezh2 (640638) and non-specific control ASO (792169) were kindly provided by Ionis Pharmaceuticals Inc (Carlsbad, CA). Matrigel Basement membrane Matrix (BD 354234) was purchased from BD Biosciences. The secondary fluorescence antibodies (Alexa Fluor 488 and Alexa Fluor 594) were purchased from Thermo Fisher Scientific.

### Chromatin immunoprecipitation (ChIP) assay

The ChIP assay was performed as described previously 39. The soluble chromatin was incubated with 2 μg of non-specific control IgG, EZH2, H3K27me3 or H3K27ac antibodies. PCR was performed using specific primers for the EZH2 binding region in the *FOXO1* promoter. Quantitative real-time PCR was performed with ChIP samples using the iQ SYBR Green Supermix and an iCycler iQTM detection system (Bio-Rad) according to manufacturer's instructions. The primer sequences are listed in Table S2.

### Quantitative reverse transcription PCR (RT-qPCR)

Total RNA was isolated with Trizol reagent (Thermo Fisher Scientific). The cDNA was synthesized using SuperScript II reverse transcriptase (Thermo Fisher Scientific). RT-qPCR was performed by using the iQ SYBR Green Supermix and an iCycler iQTM detection system (Bio-Rad) according to manufacturer's instructions. The 2-ΔΔCt method was used to calculate the relative mRNA expression level by normalizing to glyceraldehyde-3-phosphate dehydrogenase (*GAPDH*) levels. The primer sequences were listed in Table S2.

### Western blot analysis

Protein samples were prepared by lysing cells in RIPA buffer supplemented with 1% protease inhibitor cocktail (Sigma-Aldrich). The concentration of samples was measured by BCA assay kit (Thermo Fisher Scientific). Equal amounts of protein (50-100 μg) from cell lysates were denatured at 95 ^o^C for 10 mins, prior to sodium dodecyl sulfate-polyacrylamide gel electrophoresis (SDS-PAGE) and transferred to nitrocellulose membranes (Bio-Rad). The membranes were immunoblotted with specific primary antibodies (diluted at 1:1,000 to 1:5,000) at 4^ o^C overnight, followed by horseradish peroxidase (HRP) conjugated secondary antibody (diluted at 1:5,000) incubation at room temperature for 1 h. The protein signals were visualized by SuperSignal West Pico Stable Peroxide Solution (Thermo Fisher Scientific).

### MTS assay

Cells were plated in 96-well plates at a density of 3,000 cells per well for 48 h. At the indicated time points, 20 μl of MTS (CellTiter 96® AQueous One Solution reagent, Promega) was added to cells; after incubating at 37 °C for 60 min, cell viability was measured in a microplate reader at 490 nm wavelength.

### Hematoxylin-eosin (H&E) staining, immunohistochemistry (IHC) and immunofluorescent cytochemistry (IFC)

Four micrometer-thickness sections were cut from formalin-fixed paraffin-embedded (FFPE) human prostate tissues and mouse xenograft blocks. For H&E staining, the slides were deparaffinized with xylene and rehydrated through sequential ethanol washes (100% > 95% > 80% > 70%). Subsequently, slides were stained with hematoxylin, and washed with water followed by ethanol before counterstaining with 1% eosin. At last, the slides were dehydrated through graded ethanol washes and xylene washes before coverslips were sealed over the tissue sections.

For IHC, antigen retrieval and immunostaining was performed as described previously 40. Antibodies for EZH2 (1:500 dilution), FOXO1 (1:200 dilution), Ki67 (1:10,000 dilution) and cleaved Caspase-3 (1:1,000 dilution) were incubated at 4 °C overnight. The color was developed with Signal Stain ® DAB Substrate Kit.

IFC was performed as previously described 27. Cells were rinsed once with PBS and then fixed in 4% paraformaldehyde for 15 min, and washed in PBS for three times. Fixed cells were permeabilized with 0.2% Triton X-100 for 20 min, washed in PBS, and blocked in PBS supplemented with 5% goat serum and 10% glycerol. Cells were incubated with indicated primary antibodies (E-cadherin 1:500 dilution and FOXO1 1:200 dilution) at 4 °C overnight. On the second day, cells were washed three times with PBS and incubated with secondary antibody (1:500 dilution) that was conjugated with Alexa Fluor 594 and 488 (Thermo Fisher Scientific) at room temperature for 1 h. After two-time washes with PBS, the cells were counterstained with DAPI (4′, 6-diamidino-2-phenylindole) (Vector Laboratories). Images were captured using Zeiss laser confocal microscope (LSM780).

### Microscopic observations and analysis

IHC and H&E staining were observed with a Leica light microscope (10X, 20X, and 40X). IHC staining intensity and percentage for each slide was graded through unified standards. Staining intensity was graded into four categories: 0, 1, 2, and 3, of which 0 represented no staining, 1 represented low staining, 2 represented medium staining, and 3 represented strong staining. The final staining index (SI) score for each slide was obtained by multiplying staining percentage and intensity and used the SI score for correlation analysis.

### Three dimensional (3D) culture

22Rv1 or C4-2 cells stably infected with lentivirus expressing shNS or shPTEN were used for 3D culture as previously described 41. Briefly, 120 µl of Matrigel was pre-coated onto the wells of a 24-well plate for 30 min at 37 ^o^C, which is called the “first layer”. 2 x 10^4^ cells were resuspended with 250 µl of DMEM/F12 medium and seeded on the top of the first layer. After 30 min, when the cells were settled down, they were covered with the “second layer” of 10% Matrigel diluted with DMEM/F12 medium. The medium was changed with 500 µl of fresh and warm DMEM/F12 medium plus 5% FBS every 2 to 3 days. GSK126 was added to 3D cultures after two-day culture. The diameter of 3D clones was measured by Leica Application Suite software (Leica Microsystems) after 5-day treatment.

### Mouse xenograft studies

The 6-week-old NOD-SCID IL-2-receptor gamma null (NSG) male mice were generated in house for experiments. The 5 × 10^6^ C4-2 cells infected with lentivirus expression shRNA negative control or FOXO1-specific shRNA pool were prepared in 50 μl PBS plus 50 μl Matrigel and injected subcutaneous injection (s.c.) into the left flank. Approximately 15 days after injection, tumors reached the size of ~100 mm^3^, mice with shNS- or shFOXO1-expressing tumors were randomly divided into four treatment groups, with 6 mice in each group. Mice were treated intraperitoneally (i.p.) twice a week (the 1^st^ and 4^th^ day of a week) with vehicle (5% DMSO, 30% PEG 300 and 5% Tween 80) plus negative control ASO, EZH2-specific ASO at 50 mg/kg in 0.9% saline), DTX (10 mg/ml, Sandoz Inc.) at 5 mg/kg plus control ASO treatment or DTX together with EZH2-specific ASO.

For the GSK126 treatment study, mice were inoculated with C4-2 cells expressing shNS and shFOXO1 as described above. The tumor-bearing mice were randomly divided into 4 groups for both shNS and shFOXO1 genotypes. Mice were treated i.p. twice a week for 21 consecutive days with vehicle (20% Captisol, pH 4 - 4.5 plus 5% DMSO, 30% PEG 300 and 5% Tween 80), 50 mg/kg GSK126 (in 20% Captisol), 5 mg/kg of DTX (in 5% DMSO, 30% PEG 300 and 5% Tween 80) and the combination of GSK126 and DTX.

The tumor growth was measured externally by callipers and recorded twice a week. The volume of tumor was calculated using the formula 0.5 × Length (L) × Width (W)^ 2^. When the first tumor reached a volume of 1,000 mm^3^, the treatment was terminated and tumors were harvested. Each tumor was weighted and divided into three portions: one portion was used for H&E and IHC, and the other two were for protein extraction and RNA extraction, respectively. The protocol for conducting this mouse xenograft experiment was approved by Mayo Clinic IACUC.

### Graphs and statistical analysis

Graphs for RT-qPCR analysis were generated using GraphPad Prism 8.0. Experiments were performed with three or more replicates. Numerical data are presented as mean± SEM. Statistical analyses were performed by unpaired t-test. The *P* values for heat map and correlation between EZH2 and FOXO1 IHC staining were generated by R software (version 2.15.0 from http:// www.r-project.org). The correlation between EZH2 and FOXO1 is performed by Spearman's rank correlation test. Values with *P*<0.05 or smaller are considered as statistically significant. The following symbols were used to denote statistical significance: * *P*<0.05, ** *P*<0.01, *** *P*<0.001.

## Results

### *FOXO1* is a repression target gene of EZH2

**Figure 1:**
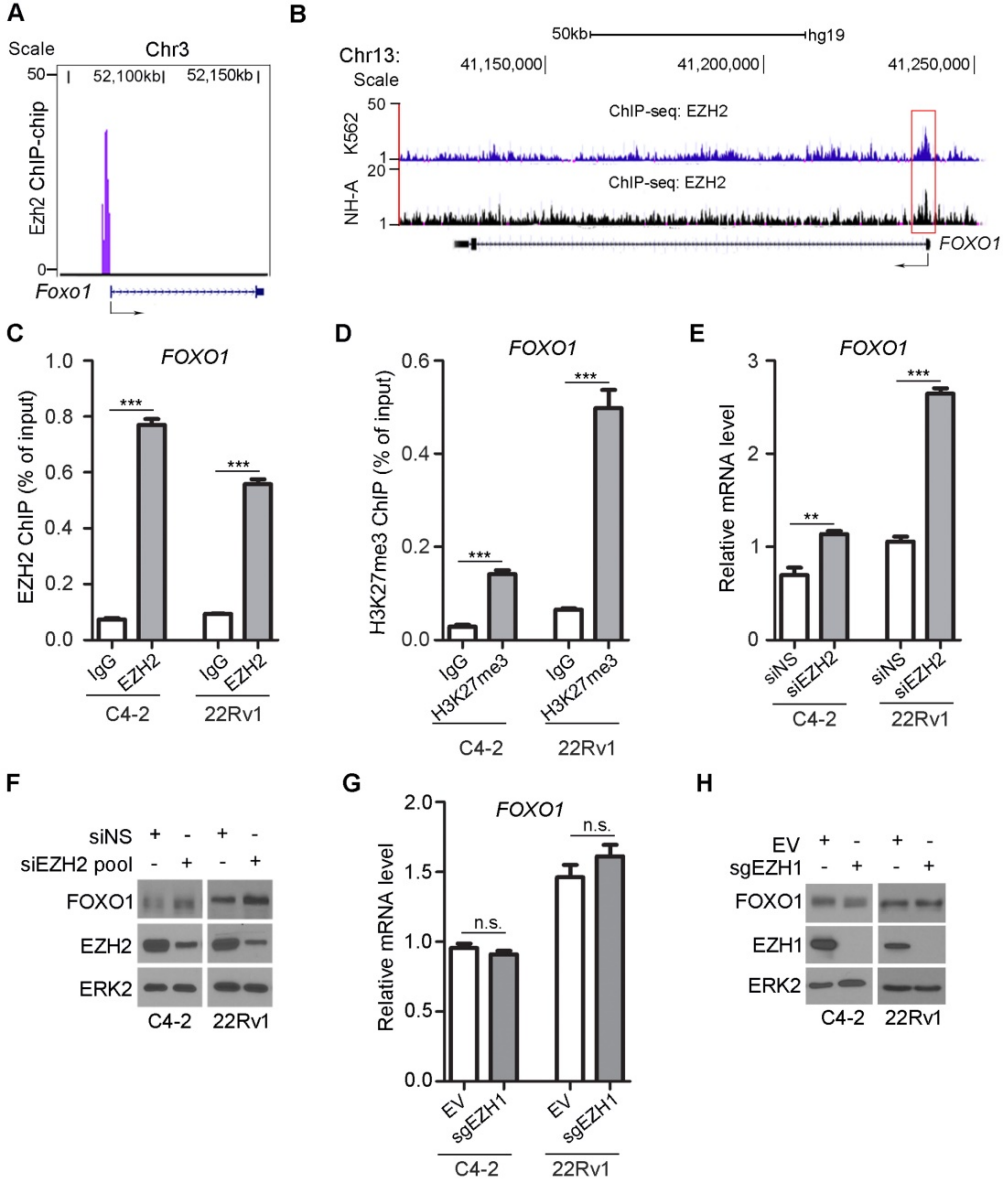
***FOXO1* gene is a repression target of EZH2. (A)** EZH2 ChIP-on-chip assay reveals murine Ezh2 binds to the *Foxo1* promoter in mouse embryo stem cells. **(B)** Screen shot of the UCSC genome browser showing ChIP-seq (reported previously 43, 44) signal profiles of EZH2 binding in the *FOXO1* gene locus in different human cell lines. **(C)** ChIP-qPCR analysis of EZH2 occupancy in the *FOXO1* promoter in both C4-2 and 22Rv1 prostate cancer cell lines. **(D)** ChIP-qPCR analysis of H3K27me3 enrichment in the *FOXO1* promoter in prostate cancer cell line C4-2 and 22Rv1 cells. **(E)** RT-qPCR analysis of *FOXO1* mRNA expression in C4-2 and 22Rv1 cells transfected with non-specific (NS) control or a pool of EZH2-specific siRNA for 48 h. RT-PCR for *GAPDH* was utilized as an internal control. **(F)** Western blot analysis of FOXO1 and EZH2 proteins in C4-2 and 22Rv1cells transfected with non-specific (NS) control or a pool of EZH2-specific siRNA for 48 h. ERK2 was used as a loading control. **(G, H)** RT-qPCR **(G)** and western blot **(H)** analysis of *FOXO1* mRNA and protein expression in C4-2 and 22Rv1 cells transfected with empty vector or EZH1-specific sgRNA and selected with puromycin for one week. RT-qPCR for *GAPDH* was utilized as an internal control. Data are shown as means ± SEM. The *P* value was performed by the unpaired two-tailed Student's t-test. * *P*<0.05; ** *P*<0.01; *** *P*<0.001; n.s., no significance.

By analyzing our genome-wide Ezh2 ChIP-on-chip dataset in mouse embryonic stem cells 42, we identified *Foxo1* as a putative Ezh2 binding target (Figure 1A). Meta-analysis of published EZH2 ChIP-seq datasets confirmed that there was an EZH2-binding peak near the transcription start site at the promoter of the *FOXO1* gene in human erythroleukemic K562 cells 43 and normal human astrocyte NH-A cells 44 (Figure 1B). ChIP-qPCR analysis further confirmed that there was substantial enrichment of EZH2 binding at the *FOXO1* promoter in both C4-2 (PTEN-negative) and 22Rv1 (PTEN-positive) prostate cancer cells (Figure 1C), indicating that EZH2 binds to the *FOXO1* gene promoter in both cell lines. As a histone methyltransferase, EZH2 is specifically involved in covalent modification of histone tails by catalyzing H3K27me3, a repressive mark enriched at the promoter of many genes that are transcriptionally silenced 45. Enrichment of H3K27me3 at the *FOXO1* promoter was also confirmed by ChIP-qPCR in these two cell lines (Figure 1D). These results support the notion that EZH2 binds to the *FOXO1* gene promoter and may repress *FOXO1* gene expression through inducing H3K27me3.

To experimentally verify whether FOXO1 is a repression target of EZH2, we examined the effect of EZH2 on FOXO1 expression at both mRNA and protein levels. EZH2 was knocked down by a pool of small interfering RNAs (siRNAs) in C4-2 and 22Rv1 prostate cancer cell lines. EZH2 depletion resulted in a significant increase in mRNA expression of *FOXO1* and two known EZH2 target genes *DAB2IP* and *BRACHYURY* in both C4-2 and 22Rv1 cell lines (Figure 1E, S1A, S1B). EZH2 regulation of *FOXO1* mRNA expression was observed in other two prostate cell lines (LNCaP and BPH1) (Figure S1C). Knockdown of endogenous EZH2 also increased the expression of FOXO1 protein in all four prostate cell lines examined (Figure 1F, S1D). In contrast, mRNA expression of other two FOXO family members *FOXO3* and *FOXO4* (*FOXO6* was not examined since its expression is neuronal specific) was unaffected by EZH2 knockdown in C4-2 and 22Rv1 cell lines (Figure S1E, S1F). Different from EZH2, depletion of EZH1 by CRISPR/Cas9 resulted in little or no effect on FOXO1 expression at both mRNA and protein level (Figure 1G, 1H). These data reveal that EZH2 specifically represses mRNA and protein expression of FOXO1, but not FOXO3 and FOXO4 in various prostate cell lines examined.

### EZH2 expression inversely correlates with FOXO1 level in prostate cancer patient specimens

**Figure 2:**
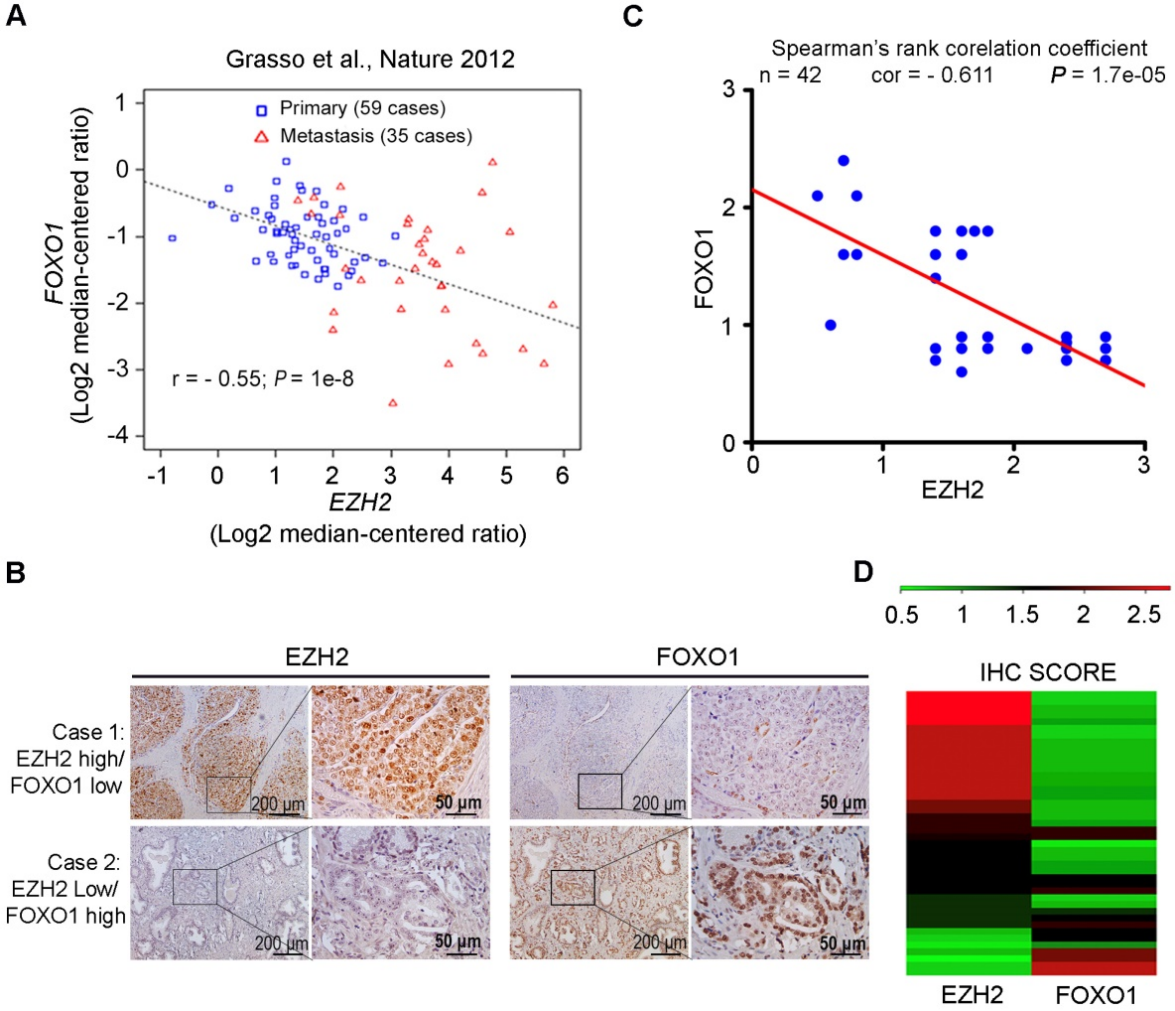
** EZH2 expression inversely correlates with FOXO1 level in prostate cancer patient specimens. (A)** Correlation analysis of *EZH2* and *FOXO1* mRNA expression in a cohort of primary (n = 59) and metastatic (n = 35) prostate cancer specimens reported previously 46. **(B)** Representative images of IHC staining of EZH2 and FOXO1 antibodies on prostate cancer patient specimens (n = 42). Scale bar in 10 X fields: 100 μm; Scale bar in 40 X fields: 20 μm. **(C)** Correlation analysis of IHC staining of EZH2 and FOXO1 proteins in prostate cancer patient specimens (n = 42).** (D)** Heat map showing IHC score (see calculation details in Materials and Methods) of EZH2 and FOXO1 protein staining on prostate cancer tissues.

Oncomining of previously reported RNA-seq data generated from prostate cancer clinical specimens 46 showed that EZH2 expression negatively correlated with FOXO1 at the mRNA level in a cohort containing both primary and metastasis prostate cancers (r = - 0.55; *P* = 1e-8) (Figure 2A). We further explored the clinical relevance of EZH2 and FOXO1 expression in an independent cohort of prostate cancer patient specimens (n = 42) using IHC. Specifically, the IHC staining was evaluated in a semi-quantitative fashion by examining both percentage of positive cells and staining intensity. Representative IHC images of high EZH2 and low FOXO1 expression and vice versa are shown in Figure 2B, respectively. In case with the high level of expression of EZH2, a relatively low level of FOXO1 expression was observed. On the contrary, in case with the low level of expression of EZH2, a relatively high level of FOXO1 was observed (Figure 2B). Further analysis showed that FOXO1 protein expression was inversely correlated with EZH2 expression in this cohort of patient specimens (Spearman's rank correlation r = - 0.611, *P* = 1.7e-05) (Figure 2C, 2D). These data suggest that EZH2 might also negatively regulate FOXO1 expression in prostate cancers in patients.

### The SET domain of EZH2 and the other core components of PRC2 complex are important for EZH2-mediated repression of FOXO1

**Figure 3:**
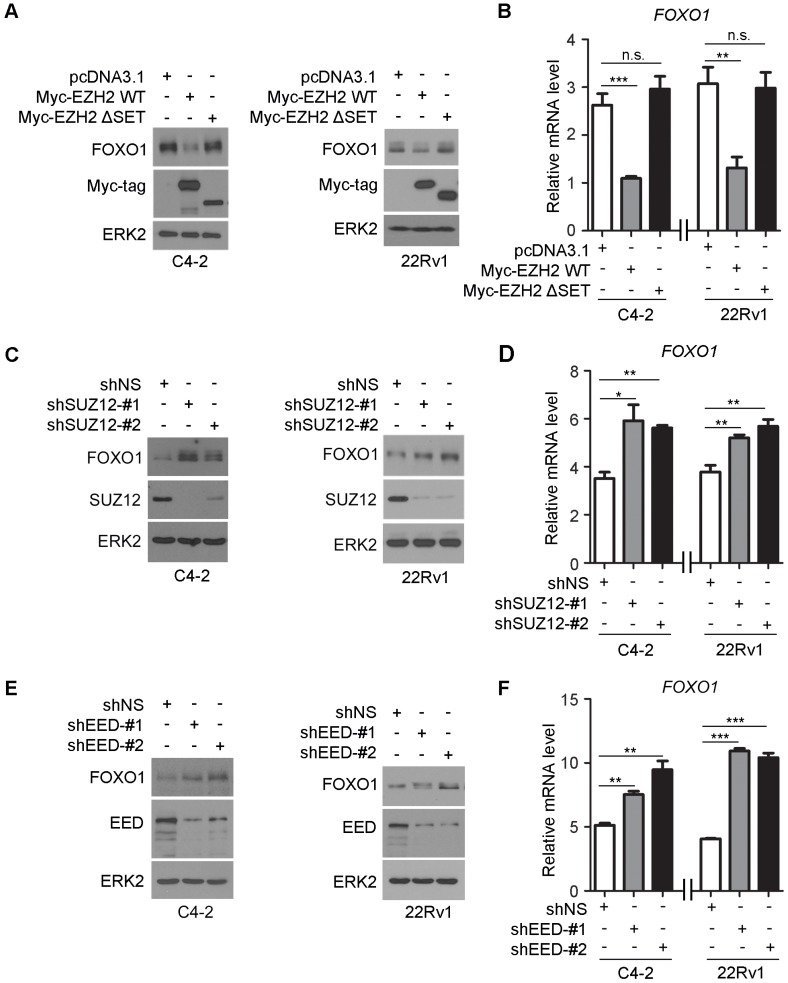
** The methyltransferase activity of EZH2 and other core components of the PRC2 complex are important for EZH2-mediated repression of FOXO1. (A, B)** C4-2 and 22Rv1 cells were transfected with the indicated plasmids for 48 h and harvested for western blot analysis **(A)** and RT-qPCR **(B)**. ERK2 was used as a loading control in western blot. *GAPDH* was utilized as an internal control in RT-PCR. **(C, D)** C4-2 and 22Rv1 cells were infected with lentivirus expressing non-specific shRNA (shNS) or SUZ12-specific shRNAs for 48 h and harvested for western blot analysis **(C)** and RT-qPCR **(D)**. **(E, F)** C4-2 and 22Rv1 cells were infected with lentivirus expressing non-specific shRNA (shNS) or EED-specific shRNAs for 48 h and harvested for western blot analysis (**E**) and RT-qPCR (**F**). Data are shown as means ± SEM. The *P* value was performed by the unpaired two-tailed Student's t-test. * *P*<0.05; ** *P*<0.01; *** *P*<0.001; n.s., no significance.

EZH2 contains a C-terminal SET domain that possesses a lysine methyltransferase activity. However, EZH2 by itself does not demonstrate any methyltransferase activity on nucleosomes. The catalytic activity of EZH2 requires the presence of at least two other core members of the PRC2 complex, namely embryonic ectoderm development (EED) and suppressor of zeste 12 (SUZ12) 3. Given that EZH2 can bind to the *FOXO1* promoter and modulate H3K27me3 in that region, we sought to determine the importance of the EZH2 SET domain in regulation of FOXO1 expression. Forced expression of wild-type EZH2, but not the SET domain truncation (ΔSET) mutant decreased FOXO1 expression at both mRNA and protein levels in C4-2 and 22Rv1 cell lines (Figure 3A, 3B). To determine the role of the other core components of PRC2 complex in EZH2 regulation of FOXO1 expression, we first identified two most effective short hairpin RNAs (shRNAs) against SUZ12 and EED. We demonstrated that knockdown of SUZ12 increased the expression of FOXO1 at both mRNA and protein levels (Figure 3C, 3D). Similar results were obtained in EED knockdown cells (Figure 3E, 3F). These data suggest that the SET domain and the other core components of PRC2 complex are important for EZH2-mediated repression of FOXO1 expression in prostate cancer cells.

### EZH2 and HDAC inhibitors upregulate FOXO1 expression

**Figure 4:**
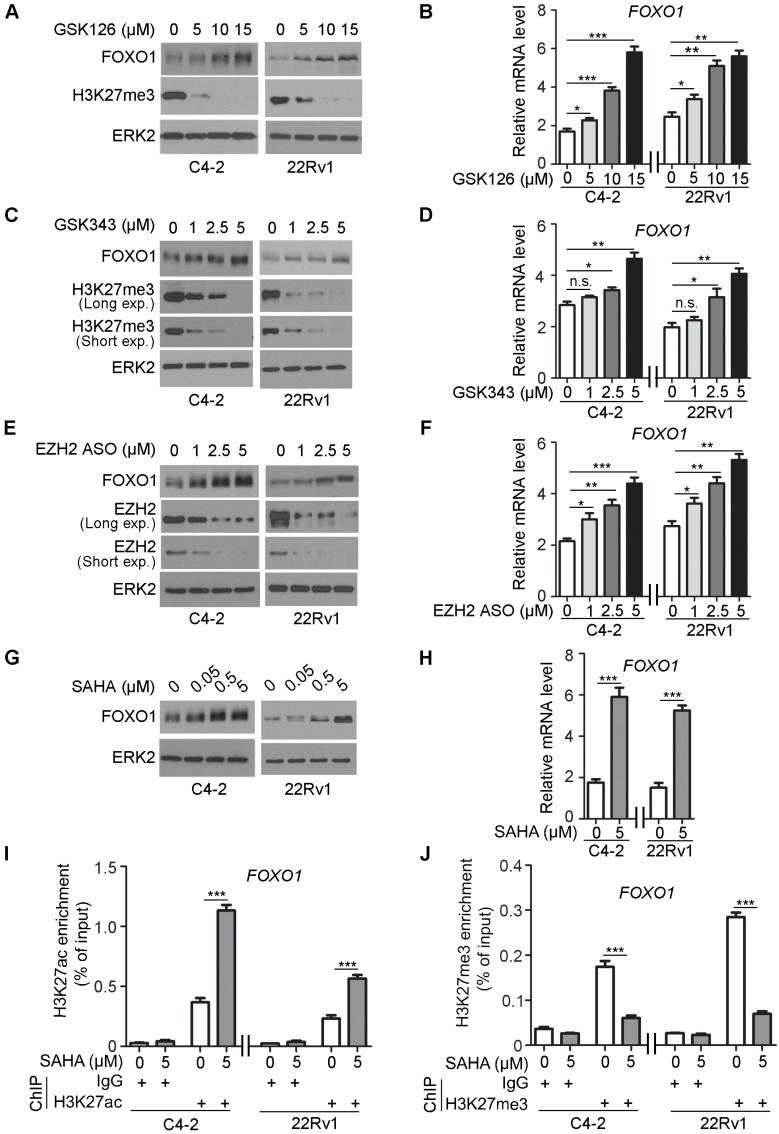
** Pharmacological inhibition of EZH2 and HDAC SAHA increase FOXO1 expression. (A, B**) C4-2 and 22Rv1 cells were treated with different concentrations of EZH2 inhibitor GSK126 for 72 h and harvested for western blot analysis **(A)** of indicated proteins and RT-qPCR analysis of mRNA expression of FOXO1 **(B)**. ERK2 was used as a loading control for western blot. *GAPDH* mRNA expression was utilized as an internal control for RT-qPCR. **(C, D)** C4-2 and 22Rv1 cells were treated with different concentrations of EZH2 inhibitor GSK343 for 72 h and harvested for western blot analysis of indicated proteins (**C**) and RT-qPCR analysis of mRNA expression of *FOXO1* **(D)**. (**E, F**) C4-2 and 22Rv1 cells were treated with different concentrations of EZH2 ASOs for 48 h and analyses were performed as in **A** and **B**. **(G, H)** C4-2 and 22Rv1 cells were treated with different concentrations of SAHA for 72 h and harvested for western blot **(G)** and RT-qPCR **(H)** analysis. **(I, J)** ChIP-qPCR analysis with H3K27ac antibody **(I)** and H3K27me3 antibody **(J)** in C4-2 and 22Rv1 cells. Data are shown as means ± SEM. The *P* value was performed by the unpaired two-tailed Student's t-test. * *P*<0.05; ** *P*<0.01; *** *P*<0.001; n.s., no significance.

Next, we employed a pharmacological approach to further determine the role of the methyltransferase activity of EZH2 in regulation of FOXO1 expression. GSK126 is a potent, highly selective, small-molecule inhibitor that inhibits EZH2 methyltransferase activity, and this compound decreases global H3K27me3 level and reactivates expression of PRC2-repressed target genes 14. C4-2 and 22Rv1 cells were treated with different concentrations (0, 5, 10 and 15 μM) of GSK126 and FOXO1 protein and mRNA level were measured. GSK126 treatment upregulated the expression of FOXO1 protein and mRNA in a dose- dependent manner in both C4-2 and 22Rv1 cell lines (Figure 4A). The efficacy of GSK126 was reflected in the depletion of H3K27me3 in these cells (Figure 4A, 4B). As a positive control, the mRNA expression of *DAB2IP*, a known EZH2 repression target gene, was also upregulated by GSK126 treatment (Figure S2A).

GSK343 is another potent, specific inhibitor of EZH2 15. C4-2 and 22Rv1 cells were treated with GSK343 (0, 1, 2.5 and 5 μM) in a manner similar to GSK126 and FOXO1 protein and mRNA level were measured. GSK343 treatment upregulated the expression of FOXO1 protein and mRNA in a dose-dependent manner in both cell lines (Figure 4C, 4D). As a positive control, *DAB2IP* displayed an increase in the mRNA level after GSK343 treatment (Figure S2B).

The antisense oligos (ASOs) have been developed as potential drugs for the treatment of human diseases including cancer 47, 48. C4-2 and 22Rv1 cells were also treated with different concentrations of EZH2 ASO (0, 1, 2.5 and 5 μM) and FOXO1 protein and mRNA level were measured. Western blot analysis showed that expression of EZH2 protein was effectively inhibited by EZH2 ASO in a dose- dependent manner (Figure 4E). Similar to the effect of GSK126 and GSK343, EZH2 ASO treatment upregulated the expression of *DAB2IP* mRNA (positive control) and FOXO1 protein and mRNA in a dose dependent manner in both C4-2 and 22Rv1 cell lines (Figure 4E, 4F, S2C).

Inhibition of deacetylases prevents the removal of the acetyl group from lysine residues of histones. Acetylated lysine residues in histones are unable to serve as substrate for methylation by EZH2 49. To further verify the regulation of FOXO1 expression by EZH2, we treated C4-2 and 22Rv1 cell lines with histone deacetylase (HDAC) inhibitor SAHA, an FDA approved drug for cancer treatment. SAHA treatment largely increased expression of *DAB2IP* mRNA (positive control) and FOXO1 mRNA and protein in C4-2 cells (Figure 4G, 4H, S2D). SAHA-induced expression of FOXO1 was consistent with the finding that SAHA treatment induced substantial increase of H3K27ac and corresponding decrease of H3K27me3 in the *FOXO1* promoter in both C4-2 and 22Rv1 cells (Figure 4I, 4J). This phenomenon was consistent with the result obtained in these cell lines treated with the EZH2 inhibitor GSK126 that inhibition of EZH2 increased H3K27ac level but decreased H3K27me3 level in the *FOXO1* promoter (Figure S2E, S2F). Similarly, in other cancer types including colorectal cancer, breast cancer and pancreatic cancer cell lines, GSK126 treatment also increased H3K27ac level but decreased H3K27me3 level in the *FOXO1* promoter (Figure S2G, S2H). Moreover, western blot analysis revealed that the treatment of GSK126 also upregulated the protein level of FOXO1 in these cancer cell lines (Figure S2I). These data suggest that histone deacetylation inhibition can achieve a comparable effect as EZH2 inhibitors in reversing EZH2-mediated repression of FOXO1 expression.

### Taxane overcomes EZH2 inhibitor resistance in PTEN-mutant cancer cells cultured *in vitro*

**Figure 5:**
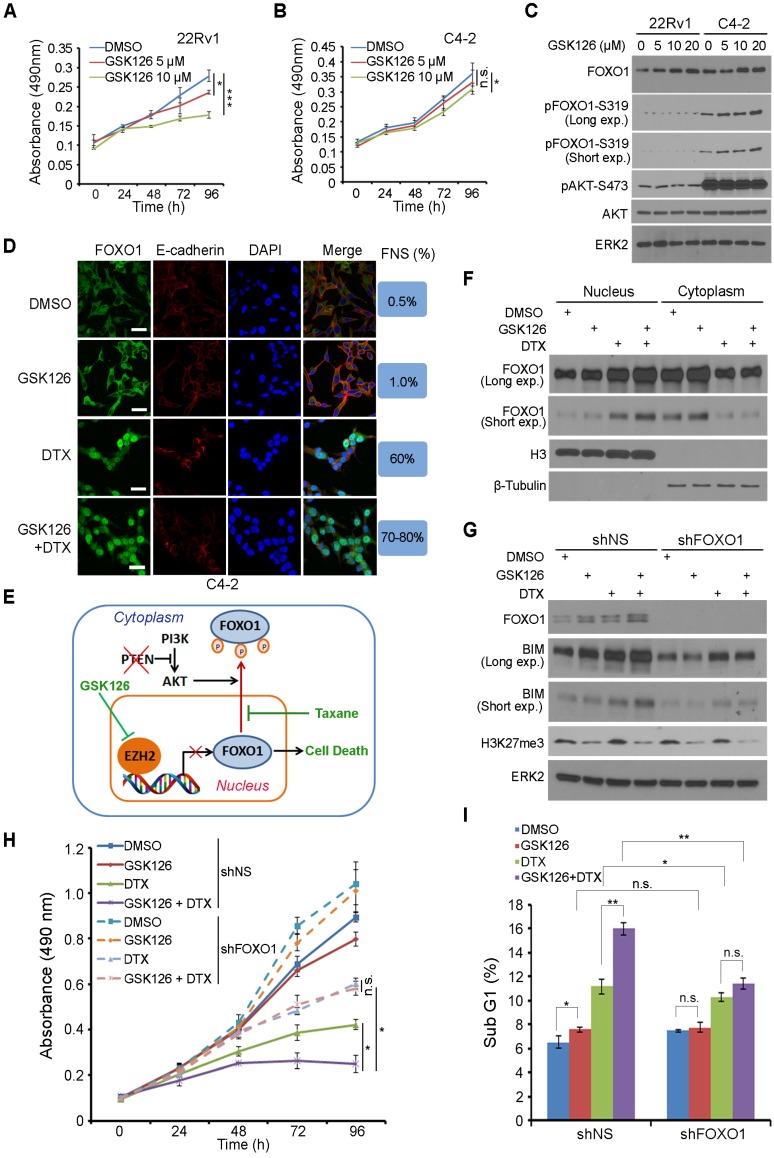
** Docetaxel overcomes EZH2 inhibitor resistance in PTEN-mutated cancer cells in culture. (A, B)** PTEN-positive 22Rv1 **(A)** and PTEN-negative C4-2 **(B)** cells were treated with different concentrations of GSK126 followed by MTS assay at different time points. **(C)** 22Rv1 and C4-2 cells were treated with different concentrations of GSK126 for 72 h and harvested for western blot analysis with the indicated antibodies. **(D)** C4-2 cells were treated with vehicle (DMSO) and GSK126 (10 μM) for 72 h followed by further treatment with or without DTX (2 nM) for 30 min prior to IFC. Cell membrane was stained with anti-E-cadherin; the nucleus was counterstained with DAPI. FNS stands for FOXO1 nuclear staining. Scale bar: 25 µm.** (E)** A hypothetical model deciphers repression of FOXO1 mRNA transcription by EZH2 and regulation of cellular localization of FOXO1 protein by taxane and the PI3K/PTEN/AKT pathway. “P” in a small red circle indicates phosphorylation. **(F)** C4-2 cells were treated with or without 10 μM of GS126 for 72 h and/or 2 nM of DTX for 30 min prior to fractionation assay and western blot analysis with indicated antibodies. Histone H3 and β-Tubulin were used as nuclear and cytosolic protein marker, respectively. **(G - I)** C4-2 cells were infected with lentivirus expressing nonspecific shRNAs (shNS) or FOXO1-specific shRNAs and selected with puromycin for stable cell lines. Cells were treated with or without 10 μM of GSK126 and/or 2 nM of DTX for 72 h and harvested for western blot analysis with indicated antibodies **(G)**, MTS assay **(H)**, and FACS analysis of percentage of sub G1 cells **(I)**. Data are shown as means ± SEM. The *P* value was performed by the unpaired two-tailed Student's t-test. * *P*<0.05; ** *P*<0.01; *** *P*<0.001; n.s., no significance.

FOXO1 functions as a key downstream effector of the PTEN tumor suppressor 24. PTEN loss leads to AKT- and CDK1- or CDK2-mediated phosphorylation of FOXO1, exclusion of FOXO1 protein from the nucleus, and loss of its tumor suppressor functions in the nucleus 18, 25-27. We sought to determine whether EZH2 regulation of FOXO1 expression plays any role in the anti-cancer effect of EZH2 inhibitor. 22Rv1 (PTEN-positive) and C4-2 (PTEN-negative) prostate cancer cell lines were treated with different doses of GSK126 and cell viability was determined by MTS assay. GSK126 treatment resulted in a dose- dependent loss of cell viability in 22Rv1 cells (Figure 5A). Surprisingly, GSK126 only induced very limited inhibition of growth in C4-2 cells (Figure 5B). To further investigate whether the status of PTEN is a determinant of drug sensitivity to the EZH2 inhibitor, we knocked down PTEN in PTEN-positive 22Rv1 and DU145 cell lines. As expected, PTEN knockdown resulted in increased AKT phosphorylation (Figure S3A). MTS assay showed that while control knockdown cells were very sensitive to GSK126 treatment, PTEN knockdown cells were much more resistant to GSK126 (Figure S3B, S3C). Furthermore, three dimentional (3D) culture studies also showed that knockdown of PTEN impaired the sensitivity of PTEN positive cells to GSK126 (Figure S3D-G). Together, these data suggest that activation of the AKT signaling might play an important role in cancer resistance to EZH2 inhibitors.

Western blot analysis showed that GSK126 treatment increased the expression of FOXO1 protein in a dose-dependent manner in both 22Rv1 and C4-2 cell lines (Figure 5C). However, different from PTEN-positive 22Rv1 cells, FOXO1 protein was highly phosphorylated by active AKT and primarily localized in the cytoplasm of PTEN-negative C4-2 cells (Figure 5C, 5D, S4A). Since both C4-2 and 22Rv1 cell lines are AR positive, we further determined whether this is the case in AR negative cell lines such as DU145 (PTEN+/-) and PC-3 (PTEN-/-). Similar to the results obtained from C4-2 and 22Rv1 cell lines, GSK126 treatment largely inhibited the growth of PTEN-positive DU145 cells but had limited effect on the growth of PTEN-null PC-3 cells (Figure S4B, S4C), and these findings are consistent with the western blot result that FOXO1 protein was highly phosphorylated by active AKT in PC-3 cells (Figure S4D). Thus, it is conceivable that EZH2 inhibitor can reverse EZH2-mediated repression of FOXO1 expression in both PTEN positive and negative prostate cancer cells; however, it induces substantial death only in PTEN-positive cells where FOXO1 protein is primarily located in the nucleus.

Docetaxel (DTX) is a semisynthetic analogue of paclitaxel, and both belong to the taxane family of chemotherapeutic drugs widely used for treatment of human cancers. We and others have shown previously that taxane treatment induces nuclear localization of FOXO proteins in AKT-active ovarian, breast and prostate cancer cells 50-52. Based upon our findings in this study and previous reports, we hypothesized that co-treatment with GSK126 and taxane can induce robust death even in PTEN-negative cancer cells through increasing FOXO1 expression (by GSK126) and nuclear localization (by taxane) (Figure 5E). To test this hypothesis, we examined cellular localization of FOXO1 in PTEN-negative C4-2 cells and PTEN-positive 22Rv1 cells using IFC and cellular fractionation assays. In support of our hypothesis, GSK126 treatment increased expression of FOXO1 protein in both C4-2 and 22Rv1 cells, but mainly in the cytoplasm of C4-2 cells (Figure 5D, 5F). Most importantly, co-treatment with GSK126 and DTX induced a robust increase of FOXO1 protein in the nucleus of both cell lines (Figure 5D, 5F, S4A).

Next we examined whether upregulation and nuclear localization of FOXO1 play any roles in the death of PTEN-negative cancer cells co-treated with GSK126 and DTX. To this end, endogenous FOXO1 was knocked down by a pool of FOXO1-specific shRNAs in PTEN-null C4-2 cells followed by drug treatment. While GSK126 alone was able to induce FOXO1 protein expression, it failed to induce high level expression of FOXO1 downstream target BIM, a pro-apoptotic protein to compromise the viability of C4-2 cells (Figure 5G, 5H). In a striking contrast, co-treatment of cells with GSK126 and DTX resulted in much greater upregulation of BIM expression and a much higher rate of growth inhibition in C4-2 cells (Figure 5G, 5H). Most importantly, the effect of the co-treatment on BIM protein expression and cell viability loss was largely diminished by FOXO1 depletion (Figure 5G, 5H). Fluorescence activated cell soring (FACS) analysis indicated that co-treatment of GSK126 and DTX induced a significant increase of cell death in C4-2 cells, but this effect was impeded by FOXO1 knockdown (Figure 5I). While GSK126 and DTX alone induced substantial loss of viability in PTEN-positive 22Rv1 cells, co-treatment of GSK126 and DTX resulted in much greater inhibition of cell viability (Figure S4E). These data indicate that DTX can overcome EZH2 inhibitor resistance in PTEN-mutant cancer cells in culture and this effect is largely mediated by expression of nuclear FOXO1.

### Co-administration of DTX and EZH2 inhibitory agents effectively inhibits PTEN-null tumor growth in mice

**Figure 6:**
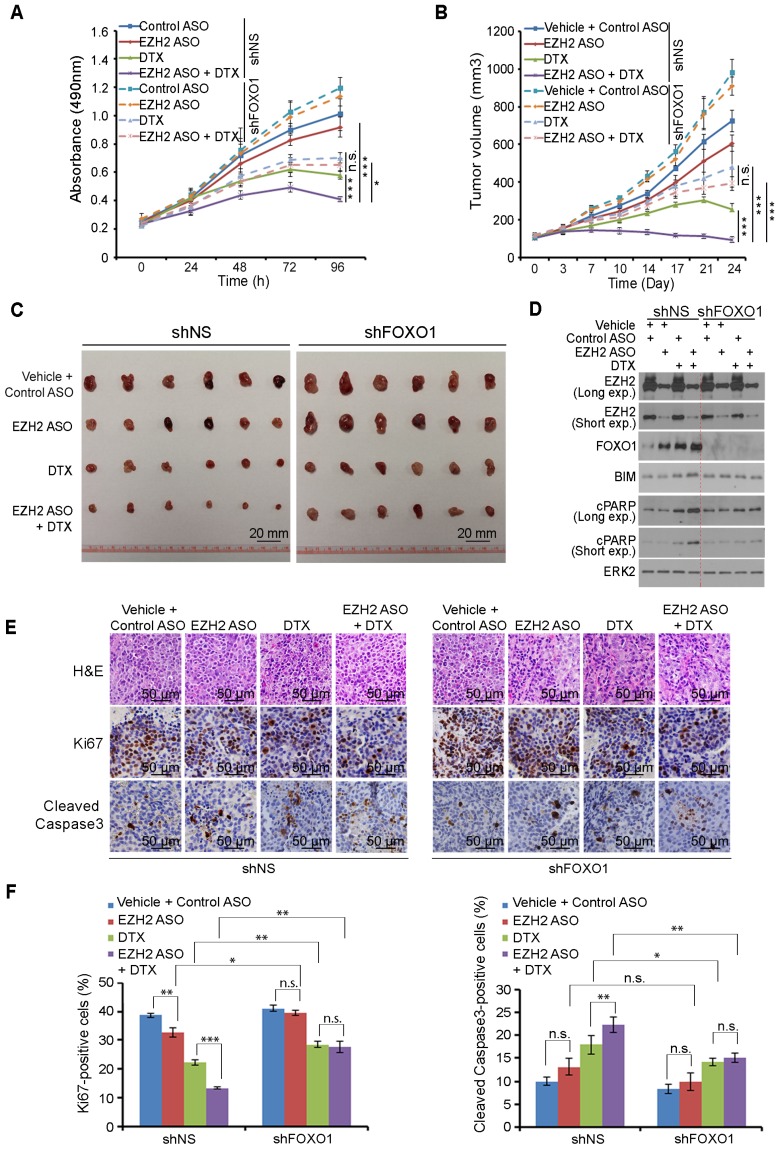
** Docetaxel overcomes EZH2 inhibitor resistance in PTEN-mutated tumors in mice. (A)** C4-2 cells were infected with lentivirus expressing non-specific shRNA (shNS) or a pool of FOXO1-specific shRNAs and selected with puromycin for stable cell lines. Cells were treated with 5 μM of control ASO or EZH2-specific ASO and/or 2 nM of DTX and MTS assay was performed at different time points. **(B, C)** C4-2 cells were infected with lentivirus expressing shNS or a pool of FOXO1-specific shRNAs as indicated and selected with puromycin. The stable cells (5×10^6^/group) were injected subcutaneously into the right flank of NSG mice. When tumors reached the size of ~100 mm^3^, mice were treated with vehicle (V) (0.9% saline) plus 50 mg/kg of control ASO, vehicle plus 50 mg/kg of EZH2 ASO, 5 mg/kg of DTX plus 50 mg/kg of control ASO or 50 mg/kg of EZH2 ASO plus 5 mg/kg of DTX by i.p. injection twice a week (the 1^st^ and 4^th^ day of week). The tumor volume at each time point was documented **(B)** and tumors in each group were harvested and photographed at day 24 **(C)**. **(D)** Western blot analysis of protein expression in xenografts harvested from mice. ERK2 was used as a loading control. The cPARP stands for cleaved PARP. **(E, F)** H&E and IHC analysis of expression of Ki-67 and cleaved Caspase-3 in xenograft sections from mice with indicated treatment. Representative images are shown in **(E)** and quantification of Ki67 and cleaved Caspase-3 positive cells from the tissue sections (n = 6) are shown in **(F)**. The number of positive cells from at least five fields were counted and analyzed. Data are shown as means ± SEM. The *P* value was performed by the unpaired two-tailed Student's t-test. * *P*<0.05; ** *P*<0.01; *** *P*<0.001; n.s., no significance.

EZH2 ASO acts similarly as GSK126 in inhibition of EZH2-mediated repression of FOXO1 expression in both C4-2 and 22Rv1 cells (Figure 4A-4F). In addition, gene-specific ASOs have been used for the treatment of human diseases in clinic 53, 54. We therefore sought to determine whether EZH2 ASO can act as EZH2 inhibitor to treat prostate cancer cells. Firstly, we tested the cytotoxicity of ASO in 22Rv1 and C4-2 cells and found that control ASO had little or no detectable effect on growth of both cell lines (Figure S5A, S5B). Next, we co-treated PTEN-negative C4-2 cells with EZH2-specific ASO and DTX. Co-administration of EZH2 ASO and DTX resulted in a robust inhibition of C4-2 cell growth *in vitro* and this effect was largely abolished by knockdown of endogenous FOXO1 (Figure 6A).

We further conducted *in vivo* experiments in NSG mice. Stable shNS- and shFOXO1-expressing C4-2 cells were injected subcutaneously into mice followed by treatment of EZH2 ASO, DTX or both. While EZH2 ASO treatment alone only slightly suppressed tumor growth and DTX alone only suppressed tumor growth moderately, co-treatment with EZH2 ASO and DTX resulted in much greater suppression of tumor growth compared to other treatment conditions (Figure 6B, 6C). These results were consistent with expression of the pro-apoptotic protein BIM and apoptotic marker cleaved PARP (cPARP) (Figure 6D). Further analysis indicated that EZH2 ASO and DTX co-treatment resulted in a greater inhibition of Ki-67 expression and increased expression of cleaved Caspase-3 (Figure 6E, 6F). When we knocked down endogenous FOXO1, the co-treatment effect of EZH2 ASO and DTX was largely diminished both *in vitro* and *in vivo* (Figures 6A-6F). Taken together, these results reveal that EZH2 ASO can suppress the growth of PTEN-negative tumors with limited effect through cell death induced by elevated FOXO1, but this suppressive effect can be largely augmented by DTX-induced nuclear localization of FOXO1. Similarly, co-treatment of mice with the EZH2 inhibitor GSK126 and DTX induced much greater inhibition of C4-2 tumors than GSK126 or DTX alone, and such effect was largely diminished by FOXO1 knockdown (Figure S5C, S5D). However, FOXO1 knockdown enhanced tumor growth compared with shNS groups and undermined synergistic inhibition of the combined treatment (Figure S5C and S5D). Together, the combination of EZH2 inhibitors, including small molecule inhibitors of the methyltransferase activity and EZH2-specific ASO with FOXO1 nuclear localization-inducing agents such as DTX can be a viable strategy for effective treatment of cancers, especially those with PTEN loss and/or activation PI3K/AKT.

## Discussion

Findings from a phase I clinical trial indicate that the EZH2 inhibitor is effective against multiple types of hematological malignancies and advanced solid tumors, and phase II studies with the EZH2 inhibitors are ongoing 55. However, the molecular mechanisms by which EZH2 inhibitors inhibit the growth of cancer cells are not fully understood. In the present study, we identify *FOXO1* as a direct downstream target gene of EZH2 in prostate cancer and other cancer types. We provide evidence that EZH2 binds to and increases H3K27me3 level at the *FOXO1* promoter and represses *FOXO1* gene expression at the transcriptional level. In addition, EZH2 expression negatively correlates with FOXO1 protein level in both cultured cancer cell lines and prostate cancer patient specimens. Most importantly, we identified FOXO1 as a key mediator of EZH2 inhibition-induced death of prostate cancer cells. Thus, our findings uncover a molecular module that links the role of FOXO1 to the action of EZH2 inhibitor in cancer.

Increasing evidence suggests that FOXO1 functions as a tumor suppressor 23. In agreement with these findings, FOXO1 expression is frequently downregulated during initiation and progression of prostate cancer 23, 56. The overexpression of EZH2 is often associated with cell proliferation and invasiveness in prostate cancer 13. While several mechanisms have been proposed for the downregulation of FOXO1, our data uncover a novel mechanism whereby increased level of EZH2 in prostate cancer results in repression of FOXO1 expression. We provide further evidence that EZH2 represses FOXO1 transcription by binding to the *FOXO1* gene promoter and increases the level of H3K27me3, a histone mark associated with compact chromatin and gene silencing 57. In agreement with a previous report that BRCA1 acts as a pivotal suppressor of EZH2 in the PRC2 complex 42, it has been shown recently that EZH2 represses expression of FOXO3, but this regulation only occurs when EZH2 becomes hyperactivated in BRCA1-deficient breast cancer cells 58. In line with these findings, we demonstrate that EZH2 only represses expression of FOXO1, but not FOXO3 or FOXO4 in BRCA1- proficient prostate cancer cell lines.

It has been proposed that EZH2-mediated gene silencing also relies on histone deacetylase (HDAC) activity 59. In support of this notion, we demonstrated that HDAC inhibitor treatment upregulates expression of FOXO1 by increasing H3K27ac level at the *FOXO1* gene promoter. Our findings support a model wherein increased HDAC activity may lead to accelerated removal of the acetyl moiety from histone in the *FOXO1* gene locus, allowing EZH2 to catalyze methylation of H3K27 and subsequently repression of *FOXO1* gene expression. Thus, our findings suggest that histone deacetylation may be a prerequisite for EZH2-mediated repression of FOXO1.

The core components of the PRC2 complex include EZH2, EED and SUZ12. While EZH2 is the only catalytic subunit of PRC2, EED and SUZ12 are also required for the PcD function of PRC2 and H3K27me3-dependent gene silencing 60. The methyltransferase activity of PRC2 depends on the catalytic SET domain of EZH2 61. We demonstrate that both the SET domain of EZH2 and other components of the PRC2 complex including EED and SUZ12 are required for EZH2-mediated repression of FOXO1, suggesting that FOXO1 is a *bona fide* PcD target.

As EZH2 is frequently overexpressed in human prostate cancers, it becomes a very attractive therapeutic target. To our surprise, EZH2 inhibitor GSK126 only works effectively in PTEN-positive prostate cancer cells, but not so in PTEN-negative counterparts. Our further investigation reveals that FOXO1 protein was induced by GSK126 in PTEN-negative cells as equivalently as in PTEN-positive cells, but was highly phosphorylated and inactivated by active AKT and localized primarily in the cytoplasm. In agreement with previous findings that taxane treatment can induce nuclear localization of FOXO proteins in ovarian, breast and prostate cancer cells 50-52, combination of GSK126 and DTX results in a significant increase in FOXO1 protein expression in the nucleus and growth inhibition in PTEN-negative cells both *in vitro* and *in vivo* in comparison to each single treatment alone. Most importantly, such effects were largely attenuated by depletion of endogenous FOXO1. The connection between the differential anti-cancer efficacies of EZH2 inhibitor in PTEN-positive versus PTEN-negative cancer cells with FOXO1 protein induction and cellular localization further highlights the essential role of FOXO1 in EZH2 inhibitor-induced death of cancer cells.

In summary, our findings identify *FOXO1* gene as a novel repression target of EZH2. We further uncover a molecular mechanism by which EZH2 mediates transcriptional repression of *FOXO1*. We provide evidence that FOXO1 plays a very important role in cancer cell death induced by EZH2 inhibitor. Most importantly, we demonstrate that EZH2 inhibitor cannot effectively induce death in PTEN-deficient cancer cells, but this can be overcome by co-treatment with taxane. Our findings suggest that EZH2 repression of expression of the FOXO1 tumor suppressor can be targeted by EZH2 inhibitor as a monotherapy for PTEN-proficient cancers or in combination with taxane for treatment of PTEN-deficient prostate cancers.

## Supplementary Material

Supplementary figures and tables.
