# Supplementary material for: Overcoming EZH2 Inhibitor Resistance by Taxane in PTEN-Mutated Cancer
Source: Theranostics. 2019 Jul 9;9(17):5020–34. doi: 10.7150/thno.34700 (PMC6691386; doi:10.7150/thno.34700)
Supplement: Supplementary file 1 — Supplementary figures and tables. [file thnov09p5020s1.pdf]

Overcoming EZH2 inhibitor resistance by taxane in PTEN-mutated prostate cancer

Linlin Ma, Yuqian Yan, Yang Bai, Yinhui Yang, Yunqian Pan, Xiaokun Gang, R. Jeffrey Karnes, Jun Zhang, Qiubo Lv, Qiang Wu and Haojie Huang

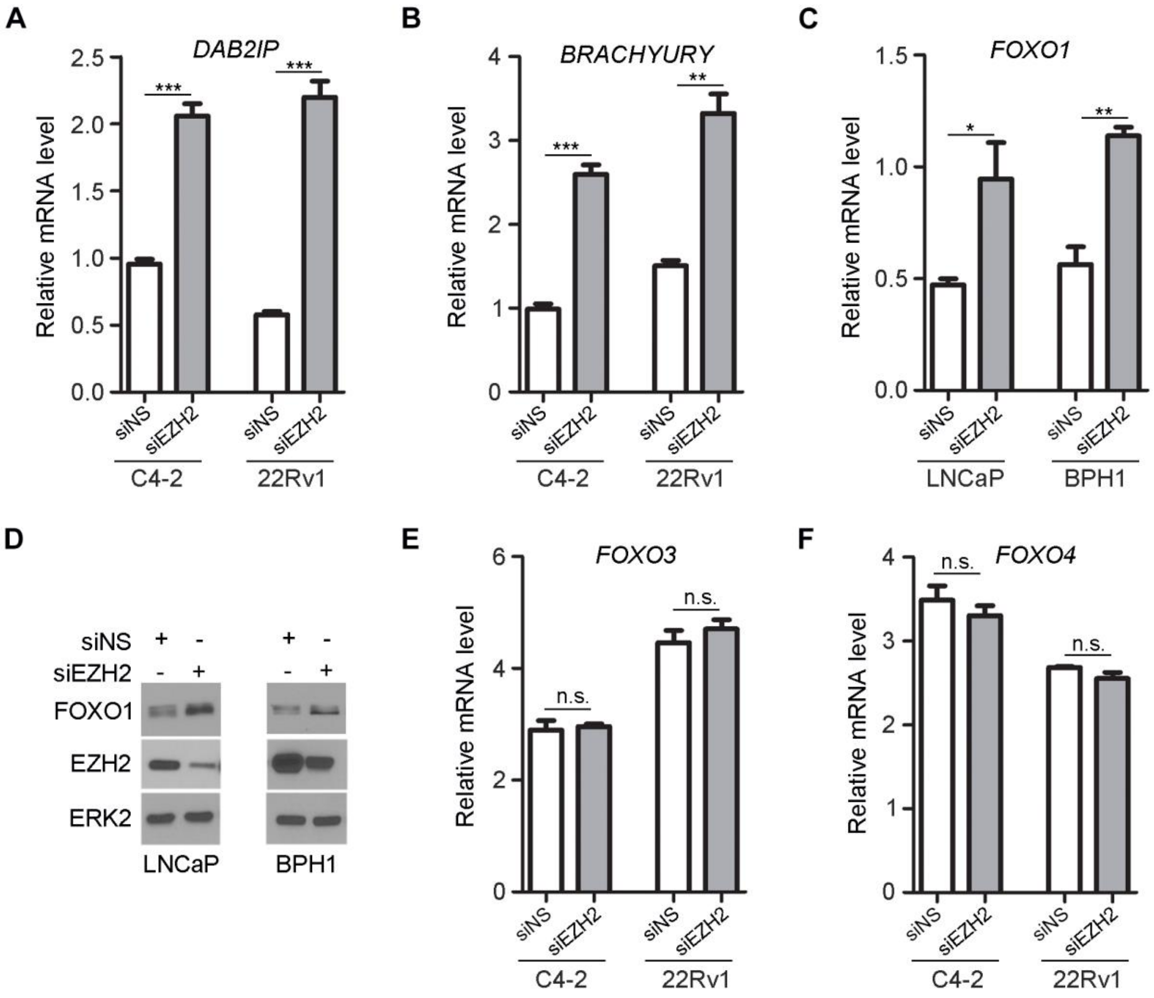

A
DAB2IP
Relative mRNA level
2.5
2.0
1.5
1.0
0.5
0.0
***
***
siNS
siEZH2
C4-2
22Rv1
B
BRACHYURY
Relative mRNA level
4
3
2
1
0
***
**
C4-2
22Rv1
C
FOXO1
Relative mRNA level
1.5
1.0
0.5
0.0
*
**
LNCaP
BPH1
D
siNS
siEZH2
FOXO1
EZH2
ERK2
LNCaP
BPH1
E
FOXO3
Relative mRNA level
6
4
2
0
n.s.
n.s.
C4-2
22Rv1
F
FOXO4
Relative mRNA level
4
3
2
1
0
n.s.
n.s.
C4-2
22Rv1

Figure S1. EZH2 represses expression of FOXO1, but not FOXO3 and FOXO4.

(A, B) RT-qPCR analysis of *DAB2IP* and *BRACHYURY* mRNA expression in C4-2 and 22Rv1 cell lines transfected with non-specific siRNA (siNS) or a pool of EZH2-specific siRNA for 48 h. **(C)** RT-qPCR analysis of *FOXO1* mRNA expression in LNCaP prostate cancer cell line and BPH1 benign prostatic epithelial cell line transfected with siNS or a pool of EZH2-specific siRNA for 48 h. **(D)** Western blot analysis of FOXO1 and EZH2 proteins in indicated cell lines transfected with siNS or a pool of EZH2-specific siRNA for 48 h. ERK2 was used as a loading control. **(E, F)** RT-qPCR analysis of expression of *FOXO3* **(E)** and *FOXO4* **(F)** mRNA in C4-2 and 22Rv1 cell lines transfected with siNS or a pool of EZH2-specific siRNA for 48 h. *GAPDH* gene was utilized as an internal control for RT-qPCR. Data are shown as means ± SEM. The *P* value was performed by the unpaired two-tailed Student's t-test. * $P<0.05$; ** $P<0.01$; *** $P<0.001$; n.s., no significance.

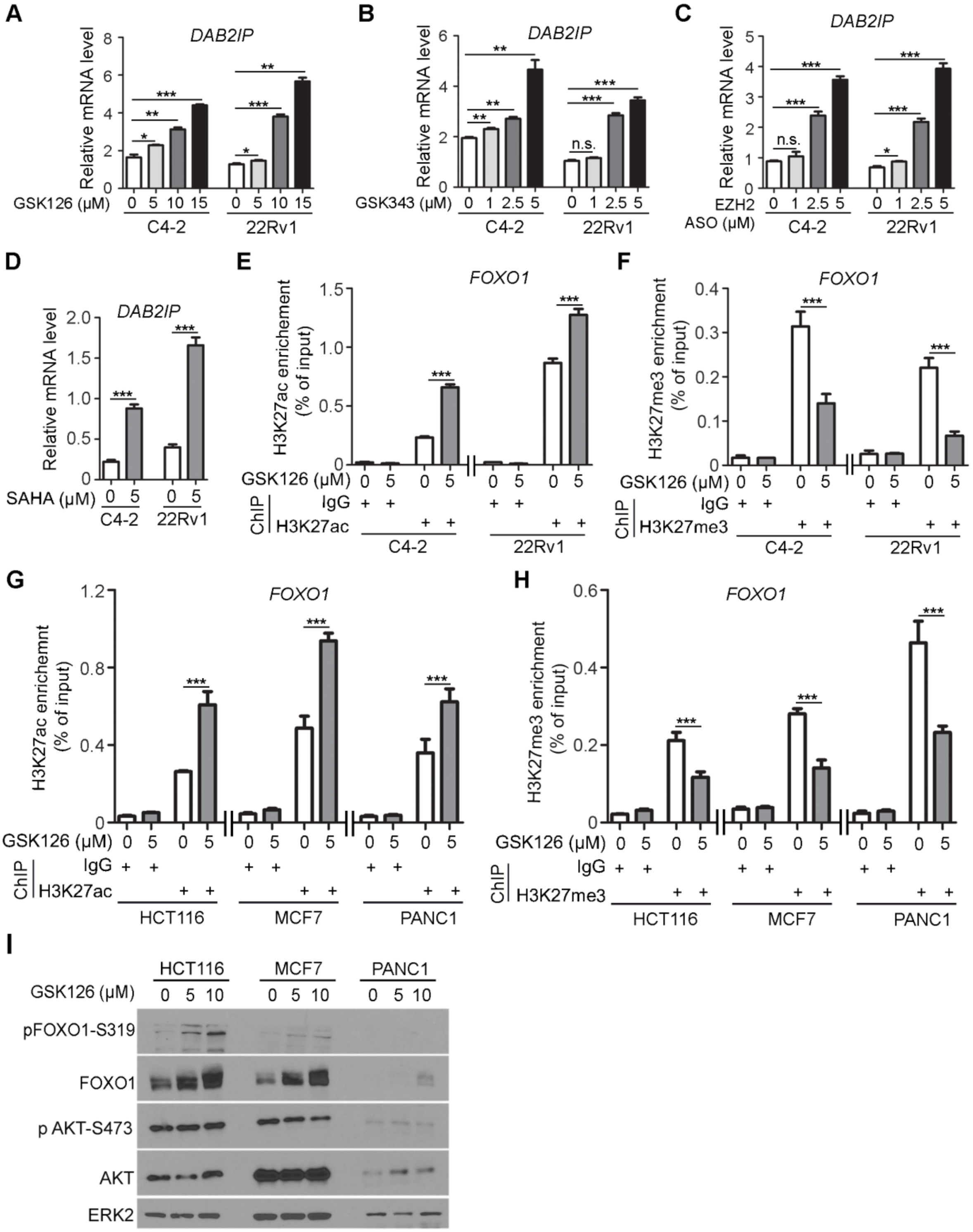
A
DAB2IP
Relative mRNA level
GSK126 (μM)
0 5 10 15 0 5 10 15
C4-2
22Rv1
B
DAB2IP
GSK343 (μM)
0 1 2.5 5 0 1 2.5 5
C
DAB2IP
n.s.
EZH2 ASO (μM)
D
SAHA (μM)
E
FOXO1
H3K27ac enrichement (% of input)
IgG
H3K27ac
ChIP
F
H3K27me3 enrichment (% of input)
H3K27me3
G
H3K27ac enrichemnt (% of input)
HCT116
MCF7
PANC1
H
I
pFOXO1-S319
FOXO1
p AKT-S473
AKT
ERK2

Figure S2. The inhibition of EZH2 and HDAC promote FOXO1 expression. (A) C4-2 and 22Rv1 cells were treated with different concentrations of EZH2 inhibitor GSK126 for 72 h and harvested for RT-qPCR analysis of mRNA expression of *DAB2IP*. **(B)** C4-2 and 22Rv1 cells were treated with different concentrations of EZH2 inhibitor GSK343 for 72 h and harvested for RT-qPCR analysis of mRNA expression of *DAB2IP*. **(C)** C4-2 and 22Rv1 cells were treated with different concentrations of EZH2 ASO for 48 h and harvested for RT-qPCR analysis of mRNA expression of *DAB2IP*. **(D)** C4-2 and 22Rv1 cells were treated with different concentrations of HDAC inhibitor SAHA for 48 h and harvested for RT-qPCR analysis of mRNA expression of *DAB2IP*. In the above experiments, *GAPDH* was utilized as an internal control for RT-qPCR. **(E, F)** C4-2 and 22Rv1 cells were treated with 2 µM of GSK126 for 48 h and harvested for ChIP-qPCR analysis with H3K27ac antibody **(E)** and H3K27me3 antibody **(F)**. **(G, H)** HCT116 (colorectal cancer), MCF7 (breast cancer) and PANC1 (pancreatic cancer) cell lines were treated with 2 µM of GSK126 for 48 h and harvested for ChIP-qPCR analysis with H3K27ac antibody **(G)** and H3K27me3 antibody **(H)**. **(I)** HCT116, MCF7 and PANC1 were treated with the indicated concentrations of GSK126 for 24 h and harvested for WB with the indicated antibodies. Data are shown as means ± SEM. The *P* value was performed by the unpaired two-tailed Student's t-test. * $P<0.05$; ** $P<0.01$; *** $P<0.001$; n.s., no significance.

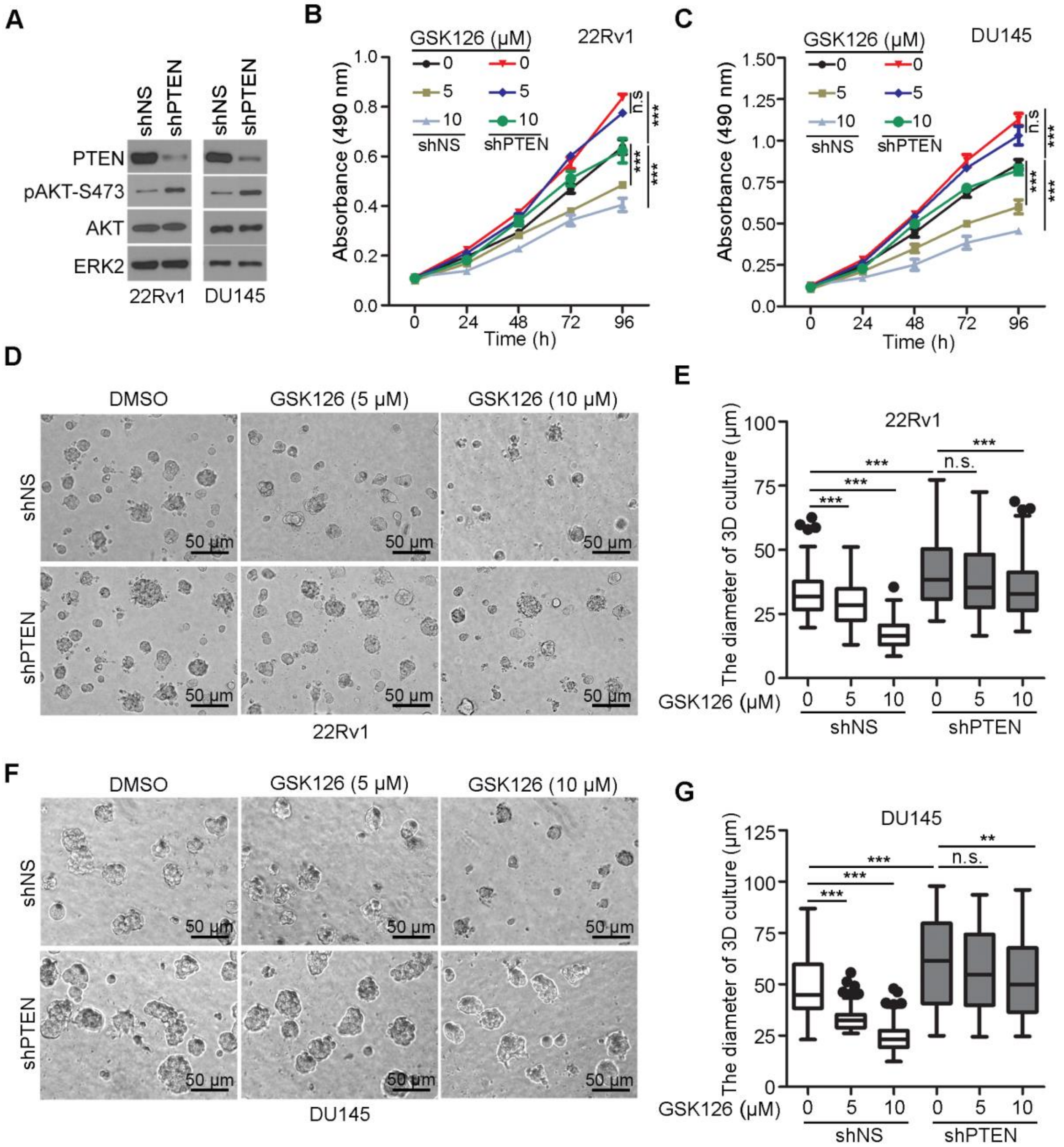
A
shNS
shPTEN
shNS
shPTEN
PTEN
pAKT-S473
AKT
ERK2
22Rv1
DU145
B
GSK126 (μM)
22Rv1
0
0
5
5
10
10
shNS
shPTEN
Absorbance (490 nm)
Time (h)
n.s
***
***
***
C
GSK126 (μM)
DU145
shNS
shPTEN
Absorbance (490 nm)
Time (h)
n.s
***
***
***
D
DMSO
GSK126 (5 μM)
GSK126 (10 μM)
shNS
shPTEN
50 μm
22Rv1
E
22Rv1
The diameter of 3D culture (μm)
n.s.
***
GSK126 (μM)
shNS
shPTEN
F
DMSO
GSK126 (5 μM)
GSK126 (10 μM)
shNS
shPTEN
50 μm
DU145
G
DU145
The diameter of 3D culture (μm)
n.s.
**
***
GSK126 (μM)
shNS
shPTEN

Figure S3. AKT signaling pathway causes the resistance to EZH2 inhibition. (A – G) 22Rv1 and DU145 cells were infected with lentivirus expressing shNS or shFOXO1 and selected with puromycin for stable cell lines. Cells were harvested to detect the transfection efficiency by western blot analysis with the indicated antibodies in **(A)** and MTS with the indicated concentrations of GSK126 in **(B)** and **(C)**. Furthermore, the infected 22Rv1 cells were plated into Matrigel for 3D culture assay with the representative images taken at day 7 **(D)** and quantification results are shown in **(E)**. Similarly, the infected DU145 cells were plated into Matrigel for 3D culture assay with the representative images taken at day 7 **(F)** and quantification results are shown in **(G)**. Data are shown as means ± SEM. The *P* value was performed by the unpaired two-tailed Student's t-test. * $P<0.05$; ** $P<0.01$; *** $P<0.001$; n.s., no significance.

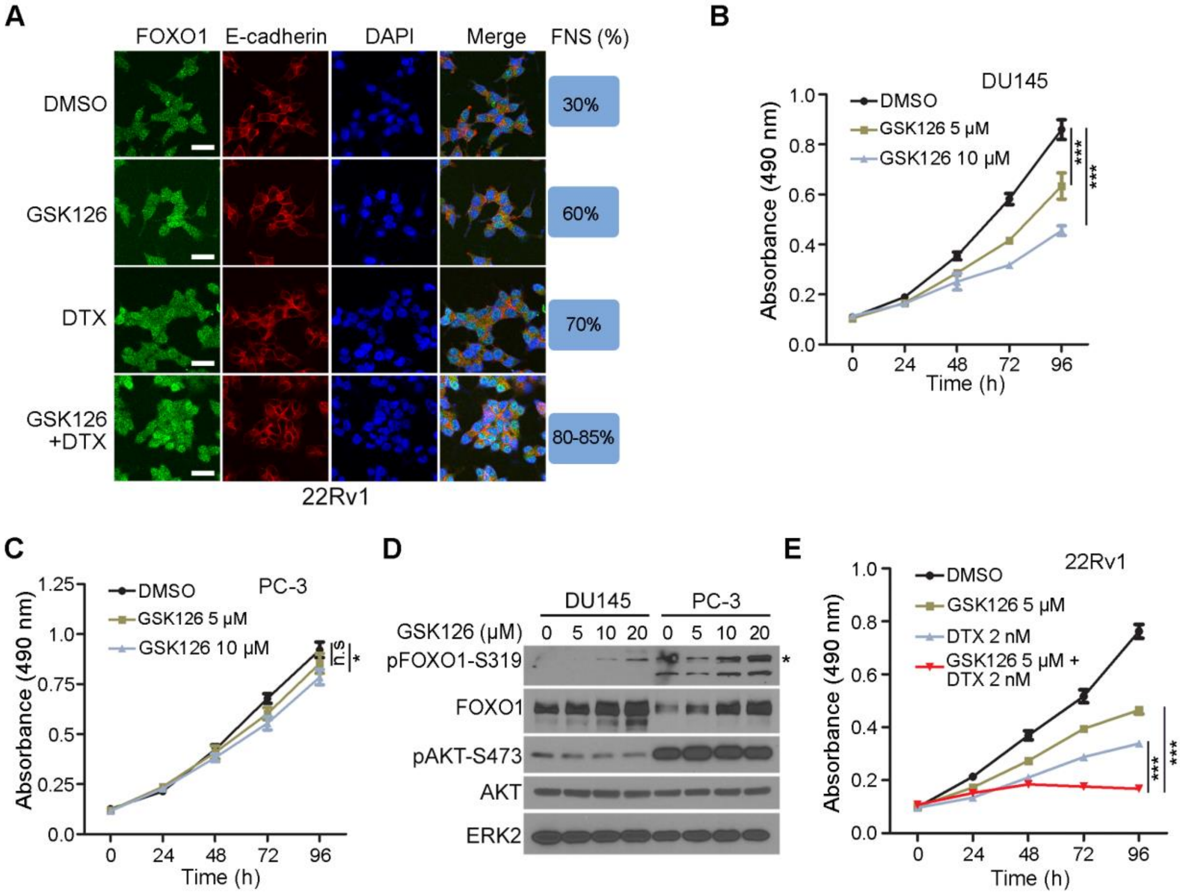
A
FOXO1
E-cadherin
DAPI
Merge
FNS (%)
DMSO
30%
GSK126
60%
DTX
70%
GSK126
+DTX
80-85%
22Rv1
B
DU145
DMSO
GSK126 5 μM
GSK126 10 μM
Absorbance (490 nm)
Time (h)
***
***
C
PC-3
DMSO
GSK126 5 μM
GSK126 10 μM
Absorbance (490 nm)
Time (h)
ns
*
D
DU145
PC-3
GSK126 (μM)
0 5 10 20 0 5 10 20
pFOXO1-S319
FOXO1
pAKT-S473
AKT
ERK2
*
E
22Rv1
DMSO
GSK126 5 μM
DTX 2 nM
GSK126 5 μM +
DTX 2 nM
Absorbance (490 nm)
Time (h)
***
***

Figure S4. Taxane sensitizes prostate cancer cells to EZH2 inhibitor. (A) 22Rv1 cells were treated with GSK126 (10 μM) for 72 h and/or DTX (2 nM) for 30 min prior to IFC. The cell membrane was co-stained with anti-E-cadherin while the nucleus was counterstained with DAPI. FNS stands for FOXO1 nuclear staining. Scale bar: 25 μm. **(B, C)** AR negative DU145 (PTEN+/-) and PC-3 (PTEN-/-) cell lines were treated with the indicated concentrations of GSK126 followed by MTS assay at different time points. **(D)** DU145 and PC-3 cells were treated with different concentrations of GSK126 for 72 h and harvested for western blot analysis with the indicated antibodies. **(E)** 22Rv1 cells were treated with GSK126 (5 μM), DTX (2 nM) and the combination of GSK126 (5 μM) and DTX (2 nM) for MTS assay at the indicated time points. Data are shown as means ± SEM. The *P* value was performed by the unpaired two-tailed Student's t-test. * $P<0.05$; ** $P<0.01$; *** $P<0.001$; n.s., no significance.

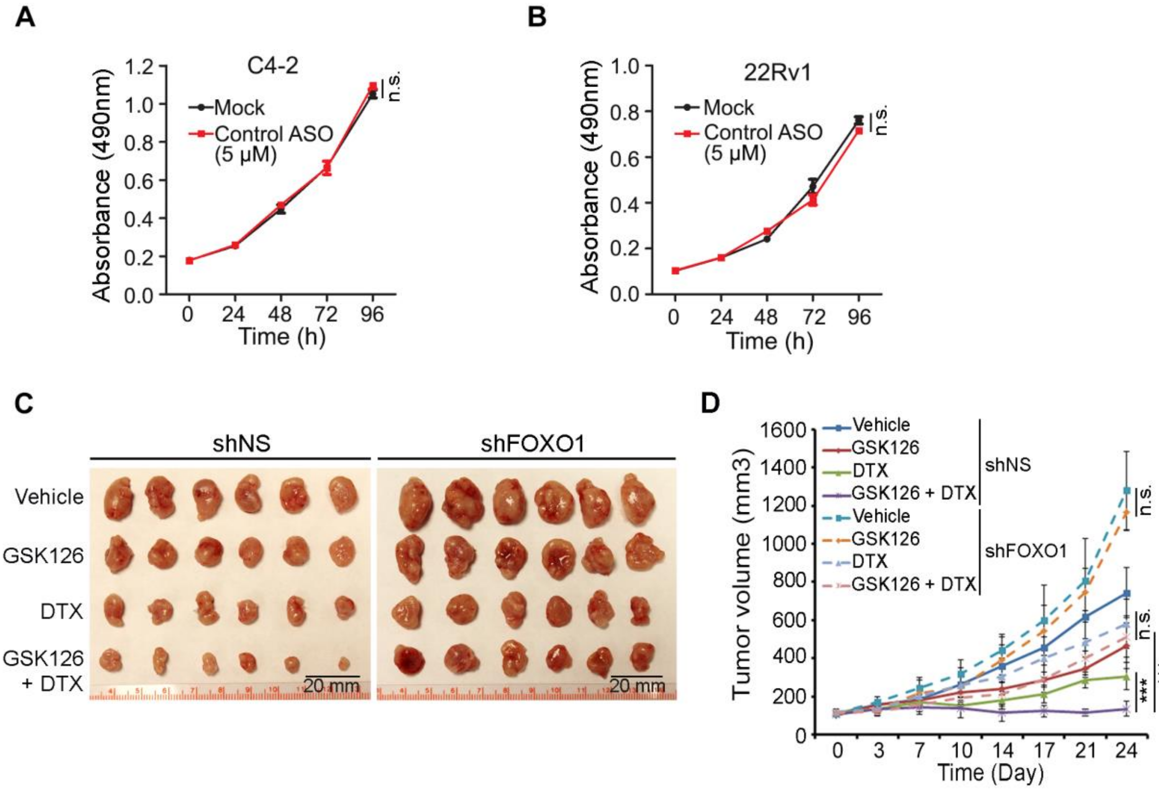
A
C4-2
Absorbance (490nm)
1.2
1.0
0.8
0.6
0.4
0.2
0.0
Mock
Control ASO
(5 μM)
n.s.
0
24
48
72
96
Time (h)
B
22Rv1
Absorbance (490nm)
1.0
0.8
0.6
0.4
0.2
0.0
Mock
Control ASO
(5 μM)
n.s.
0
24
48
72
96
Time (h)
C
shNS
shFOXO1
Vehicle
GSK126
DTX
GSK126
+ DTX
20 mm
20 mm
D
Tumor volume (mm3)
1600
1400
1200
1000
800
600
400
200
0
Vehicle
GSK126
DTX
GSK126 + DTX
shNS
Vehicle
GSK126
DTX
GSK126 + DTX
shFOXO1
n.s.
n.s.
***
***
0
3
7
10
14
17
21
24
Time (Day)

Figure S5. FOXO1 plays a critical role in the sensitivity of EZH2 inhibition. (A, B) C4-2 and 22Rv1 cells were treated with 5 μM of non-specific control ASO and subjected for MTS at the different time points. **(C, D)** Mice with C4-2 xenograft tumors were treated with vehicle (20% Captisol), GSK126 (50 mg/kg), DTX (5 mg/kg) or the combination of GSK126 (50 mg/kg) and DTX (5 mg/kg) five days a week for three consecutive weeks. The tumors isolated at day 24 are shown in **(C)** with quantification results shown in **(D)**.

Table S1. Sequences of siRNAs and shRNAs used in the manuscript.

Gene	Usage	Sequences (5’-3’)
EZH2	siRNA	GGAAAGAACUGAAACCUUA
		CAGAAGAGCUGAUGAAGUA
FOXO1	shRNA-#1	CCGGATCTACGAGTGGATGGTCAACTCGAGTTGACCATCCACTCGTAGATCTTTTTG
	shRNA-#2	CCGGGCCGGAGTTTAGCCAGTCCAACTCGAGTTGGACTGGCTAAACTCCGGCTTTTTG
EED	shRNA-#1	CCGGCCAGAGACATACATAGGAATTCTCGAGAATTCCTATGTATGTCTCTGGTTTTT
	shRNA-#2	CCGGCCAGTGAATCTAATGTGACTACTCGAGTAGTCACATTAGATTCACTGGTTTTT
SUZ12	shRNA-#1	CCGGCGGAATCTCATAGCACCAATACTCGAGTATTGGTGCTATGAGATTCCGTTTTTG
	shRNA-#2	CCGGGCTGACAATCAAATGAATCATCTCGAGATGATTCATTTGATTGTCAGCTTTTTG

Table S2. Sequences of primers used in the manuscript.

Gene	Usage	Sequences (5’ – 3’)	Sequences (5’ – 3’)
FOXO1	ChIP-qPCR	GGGGTAGTGGGGTGTTTTTC	AGTACTCGGCTCTGCTGCTC
FOXO1	RT-qPCR	TTATGACCGAACAGGATGATCTTG	TGTTGGTGATGAGAGAAGGTTGAG
FOXO3	RT-qPCR	GTTTGAACGTGGGGAACTTCACTG	AACGGTATCACTGTCCACTTGCTG
FOXO4	RT-qPCR	ACGAGTGGATGGTCCGTACT	GGTTCAGCATCCACCAAGAG
DAB2IP	RT-qPCR	TGGACGATGTGCTCTATGCC	GGATGGTGATGGTTTGGTAG
BRACHYURY	RT-qPCR	GGAGCTCACCAATGAGATGA	CGAGTCGGGGTGGATGTAGA
GAPDH	RT-qPCR	ACCCACTCCTCCACCTTTGAC	TGTTGCTGTAGCCAAATTCGTT
EZH1	sgRNA	CACCGTCGACAACTTAAACGGCTTC	AAACGAAGCCGTTTAAGTTGTCGAC
